# Angiopoietins and diabetic nephropathy

**DOI:** 10.1007/s00125-016-3995-3

**Published:** 2016-05-20

**Authors:** Luigi Gnudi

**Affiliations:** Unit for Metabolic Medicine, Cardiovascular Division, Faculty of Life Science & Medicine, King’s College London, 3rd Floor Franklin-Wilkins Building, Waterloo Campus, Stamford Street, London, SE1 9RT UK

**Keywords:** Albuminuria, Angiopoietin, Endothelial cells, Diabetes, Glomerulus, Review

## Abstract

Diabetic nephropathy is the main cause of end-stage renal failure in the Western world. In diabetes, metabolic and haemodynamic perturbations disrupt the integrity of the glomerular filtration barrier, leading to ultrastructural alterations of the glomeruli, including podocyte foot process fusion and detachment, glomerular basement membrane thickening, reduced endothelial cell glycocalyx, and mesangial extracellular matrix accumulation and glomerulosclerosis, ultimately leading to albuminuria and end-stage renal disease. Many vascular growth factors, such as angiopoietins, are implicated in glomerular biology. In normal physiology angiopoietins regulate the function of the glomerular filtration barrier. When they are dysregulated, however, as they are in diabetes, they drive the cellular mechanisms that mediate diabetic glomerular pathology. Modulation of angiopoietins expression and signalling has been proposed as a tool to correct the cellular mechanisms involved in the pathophysiology of diabetic microvascular disease, such as retinopathy in humans. Future work might evaluate whether this novel therapeutic approach should be extended to diabetic kidney disease.

## Introduction

Angiopoietins are vascular growth factors involved in vasculogenesis and vascular repair. Two major isoforms regulate vascular homeostasis, namely angiopoietin 1 (ANGPT1) and angiopoietin 2 (ANGPT2). ANGPT1, by binding to its receptor tyrosine kinase with immunoglobulin and epidermal growth factor homology domain-2 (TIE-2), stabilises the vessel wall, while ANGPT2, by either interacting with integrins or competing with ANGPT1-TIE-2 receptor binding (inhibiting ANGPT1-mediated TIE-2 phosphorylation), promotes vessel wall destabilisation and favours, in the presence of vascular endothelial growth factor A (VEGFA), endothelial cell proliferation and new vessel formation.

Angiopoietins play an important role in the glomerular capillaries in both physiology and disease. Glomerular capillaries are unique in their structure: they are composed of a fenestrated endothelium, which sits on a basement membrane, and specialised epithelial cells (the podocytes), which cover the external layers of the glomerular filter with their interdigitating foot processes.

In recent years, work by different investigators has highlighted the presence of an autocrine/paracrine network consisting of angiopoietins and other vasoactive peptides regulating the physiology of the glomerular capillaries in terms of blood flow and permeability of the vascular wall. An imbalance in these vasoactive peptides promotes endothelial dysfunction: one of the earliest mechanisms of vascular chronic complications in diabetes.

This brief review focuses on the role of angiopoietins in physiology and how an angiopoietin imbalance could contribute to the pathophysiology of diabetic glomerulopathy.

## Glomerular capillary function is regulated by vasoactive peptides within an epithelial–endothelial autocrine/paracrine system

The kidney glomerulus filters the blood to generate urine by retaining cells and macromolecules. The adult glomerulus consists of specialised capillaries composed of epithelial cells (podocytes) separated from the endothelium and mesangium by a thin glomerular basement membrane (GBM) [1]. All the components of the glomerular filtration barrier participate in the maintenance of the permselective properties of the glomerular capillaries. Podocyte cells are composed of a major cell body from which specialised foot processes depart to interdigitate and form slit diaphragms [2], a specialised tight junction (composed of proteins such as nephrin and podocin) that represents one of the major size-selective barriers in the glomerular capillaries preventing macromolecular passage into the pre-urine [3, 4]. The glomerular endothelial cells are fenestrated; the fenestrae are characterised by a unique ultrastructure lacking diaphragms [5, 6] and facilitate high permeability to water and small solutes [7]. The luminal side of the glomerular endothelial cells is covered by a thin glycocalyx consisting of proteoglycans [7], which are also believed to contribute to the permselective properties of the glomerular capillary [8]. The GBM also contributes to the permeability of the glomerular capillary, and alteration of its structure affects its ability to restrict both water [7] and protein [9] filtration through the glomerular barrier.

Many recent studies have proposed a complex local autocrine/paracrine network consisting of vascular growth factors and vasoactive peptides secreted by glomerular cells which exert their action by binding to their specific receptor on podocytes and glomerular endothelial cells. The specific balance of different growth factors such as angiopoietins and VEGFA and their interaction have been recognised as crucial mechanisms in the maintenance of a healthy glomerulus; conversely, their imbalance has been shown to drive the early pathological manifestations of diabetic glomerular disease.

## Angiopoietins

Angiopoietins are vascular growth factors that promote the formation of blood vessels. ANGPT1 and ANGPT2 are the most studied angiopoietins [10]. ANGPT1 is the major physiological ligand and activator (via phosphorylation) of the TIE-2 receptor [11, 12]. ANGPT1 is extremely important in early vasculature growth up to embryonic days 9.5–12.5 [13, 14]. Studies also suggest that ANGPT1 is important in maintaining the stability (and permeability) of the mature vasculature [15, 16]. This concept has, however, been challenged by studies in mice with inducible global deletion of *Angpt1* from embryonic day 13.5 [14], in which animals were viable and presented with no overt phenotype [14].

ANGPT2 has opposing actions to ANGPT1 and promotes blood vessel wall destabilisation [17], not only by competitive inhibition of the binding of ANGPT1 to TIE-2, hence reducing TIE-2 activation and phosphorylation [17], but also via integrin activation [18]. The biological effects of ANGPT2 appear to be dependent on the ambient levels of VEGFA: ANGPT2 leads to vessel regression when VEGFA expression/levels are low or absent, but participates in the process of angiogenesis in the presence of high levels of surrounding VEGFA [17]. Studies have shown that VEGFA inhibits ANGPT1–TIE-2 signalling via TIE-2 shedding, promoting, in concert with ANGPT2-mediated TIE-2 signalling inhibition, new vessel formation [19].

In the kidney glomerulus, *Angpt1* is constitutively expressed in podocytes [20, 21], while *Angpt2* is transiently detected during development in the mesangial cells [22], but is then not expressed, or expressed at a very low level, in the adult glomerulus in normal physiology. *Tie2* (also known as *Tek*) expression is localised in developing and adult mouse glomerular capillaries mainly at the level of the endothelium [20, 23], while some reports show its expression in mouse and rat podocytes *in vivo* [21, 24].

## Angiopoietins and diabetic glomerulopathy

The expression of angiopoietins in glomerular disease has been investigated in different experimental animal models of diabetes. In rats injected with streptozotocin, whole-kidney *Angpt1* and *Angpt2* mRNA and protein increase at 4 weeks’ diabetes duration, but after 8 weeks ANGPT1 levels diminish, while ANGPT2 remains elevated [25]. In another study, whole glomeruli or glomerular endothelial cells isolated from diabetic mice showed raised *Angpt2* mRNA levels compared with non-diabetic animals, while no changes were observed in *Angpt1* in the whole glomerulus [14].

Transgenic mouse studies have demonstrated that raised *Angpt2* leads to a phenotype similar to that seen in diabetic glomerular disease. Indeed, inducible podocyte-specific overexpression of *Angpt2*, increasing the ANGPT2/ANGPT1 ratio in otherwise normal healthy adult mice, leads to albuminuria and glomerular endothelial apoptosis, a phenotype paralleled by downregulation of VEGFA and nephrin protein expression [26]. Similarly, ANGPT2 has been shown to activate β1-integrin, resulting in destabilisation of endothelial cell intercellular junctions via an increase in cell contractility and alteration of pericellular matrix remodelling [18].

**Fig. 1:**
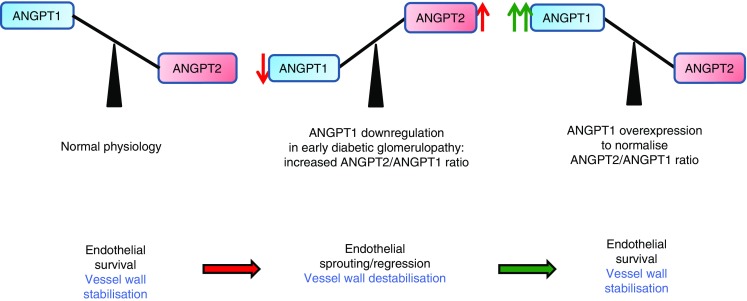
ANGPT2/ANGPT1 imbalance is paralleled by capillary destabilisation. ANGPT1 is downregulated in early diabetic kidney disease, leading to diabetes-mediated angiopoietin imbalance (ANGPT2 > ANGPT1); ANGPT2 excess results in proteinuria. Repletion of ANGPT1 restores angiopoietin balance (ANGPT1 > ANGPT2) and, acting as a ‘brake’ on vascular lesions, prevents albuminuria and glomerular structural lesions in the early phases of diabetic glomerular disease. Red arrows indicate changes favouring progression towards vascular disease, green arrows point towards changes stimulating a healthy vessel

Parallel work from our group has shown that, in the very early stages of diabetic glomerulopathy (3 weeks’ diabetes duration), glomerular *Angpt1* mRNA decreases in diabetic mice, with no significant changes in *Angpt2* mRNA levels, when compared with non-diabetic animals [24]. This apparent acute effect of elevated circulating glucose levels was also observed in vitro when *Angpt1* mRNA was significantly downregulated in high-glucose-treated podocytes compared with normal-glucose-treated cells [24]. Overall, these observations are consistent with the contention that an increased ratio of ANGPT2/ANGPT1 could play a role in the development and progression of glomerular disease in diabetes (Fig. 1).

Studies using genetically modified mice have further helped towards the understanding of angiopoietins in diabetic glomerular disease. Global deletion of *Angpt1* from embryonic day 16.5 (to circumvent any adverse effects on early vascular development) is paralleled, in mice at 20 weeks’ diabetes duration, by increases in albuminuria and mesangial matrix expansion and glomerulosclerosis [14], suggesting that *Angpt1* expression levels are important in the pathophysiology of diabetic glomerular disease and could confer protection against high-glucose-mediated glomerular capillary injury, serving as a ‘brake’ on vascular lesions.

To further explore this possibility we restored the ANGPT1 deficiency found in early diabetic glomerulopathy using transgenic mice overexpressing *Angpt1*, specifically in the glomeruli, again with an inducible system [24]. Podocyte-specific ANGPT1 overexpression/repletion in the adult diabetic mouse led to a reduction in albuminuria [24] and downregulation of diabetes-induced VEGFA signalling. The combination of high ANGPT1 levels and low VEGFA signalling in diabetic nephropathy is likely to represent an important mechanism that favours a more stable capillary wall, a phenomenon that we described as paralleled by a reduction in glomerular endothelial cell proliferation, as seen in the early stages of diabetic nephropathy [27], and a reduction in albumin excretion [24].

Overexpression/repletion of ANGPT1 in diabetic mice increased the phosphorylation of endothelial nitric oxide synthase on serine residue 1177 [24], an effect that may increase nitric oxide [28] and lead to a more stable, less permeable vascular wall [29, 30]. ANGPT1 repletion was also accompanied by a reduction in diabetes-induced nephrin phosphorylation [24], an event paralleled by reduced nephrin degradation and improved foot processes in the podocyte cytoskeleton/structure, leading to a more intact and functional glomerular filtration barrier [31].

*ANGPT2* mRNA was found to be elevated in isolated glomeruli from patients with diabetes when compared with live donor kidneys, while no change was observed in *ANGPT1* expression [24]. Importantly, poor glycaemic control is paralleled by high circulating ANGPT2 levels in patients with type 2 diabetes. ANGPT2 levels are also associated with indexes of endothelial damage/dysfunction, regardless of vascular disease [32]. Similarly, urinary ANGPT2 levels are increased in patients with type 2 diabetes with renal damage and are associated with albuminuria [33].

A recent study reported preliminary evidence that ANGPT2 is an independent predictor of adverse renal outcome in chronic kidney disease in both the general and the diabetic population [34]. Of interest, raised ANGPT2 levels are associated with systemic markers of inflammation in patients with chronic kidney disease and are predictors of mortality [35]. For example, increased aortic stiffness is known to be a powerful independent predictor of mortality in individuals with type 2 diabetes [36], and recent work has shown that plasma ANGPT2 is associated with arterial stiffness in patients with chronic kidney disease [37]. Indeed, an imbalance in favour of ANGPT2 would promote inflammation and fibrosis, with macrophages as key players in stimulating collagen-rich extracellular matrix synthesis by vascular smooth muscle cells with stiffening of the vascular wall [38].

Taking all these observations together, it is quite evident that a tightly controlled angiopoietin TIE-2 receptor system is required for the development and maintenance of a healthy microvasculature and glomerular filtration barrier. Disruption of the ANGPT2/ANGPT1 balance in favour of ANGPT2 leads to destabilisation of the capillary walls and an increase in inflammation and vascular permeability, promoting microvascular disease.

Manipulation of local and systemic angiopoietins could represent an attractive therapeutic target for patients with diabetic microvascular complications [39, 40]. Early studies in patients with diabetes with macular oedema have shown that administration of AKB-9778 (a vascular endothelial-protein tyrosine phosphatase that promotes TIE-2 receptor activation) for 4 weeks reduced macular oedema and improved vision, without demonstrating any safety concerns [41]. Future studies might address the role of TIE-2 activation in diabetic glomerular disease.

## Abbreviations

ANGPT1: Angiopoietin 1
ANGPT2: Angiopoietin 2
GBM: Glomerular basement membrane
TIE-2: Tyrosine kinase with immunoglobulin and epidermal growth factor homology domain-2
VEGFA: Vascular endothelial growth factor A

## References

[1] Tryggvason K, Pikkarainen T, Patrakka J (2006). Nck links nephrin to actin in kidney podocytes. Cell.

[2] Greka A, Mundel P (2012). Cell biology and pathology of podocytes. Annu Rev Physiol.

[3] Boute N, Gribouval O, Roselli S (2000). NPHS2, encoding the glomerular protein podocin, is mutated in autosomal recessive steroid-resistant nephrotic syndrome. Nat Genet.

[4] Kestila M, Lenkkeri U, Mannikko M (1998). Positionally cloned gene for a novel glomerular protein—nephrin—is mutated in congenital nephrotic syndrome. Mol Cell.

[5] Ichimura K, Stan RV, Kurihara H, Sakai T (2008). Glomerular endothelial cells form diaphragms during development and pathologic conditions. J Am Soc Nephrol.

[6] Brunskill EW, Potter SS (2010). Gene expression programs of mouse endothelial cells in kidney development and disease. PLoS One.

[7] Haraldsson B, Nystrom J, Deen WM (2008). Properties of the glomerular barrier and mechanisms of proteinuria. Physiol Rev.

[8] Salmon AH, Ferguson JK, Burford JL (2012). Loss of the endothelial glycocalyx links albuminuria and vascular dysfunction. J Am Soc Nephrol.

[9] Suh JH, Miner JH (2013). The glomerular basement membrane as a barrier to albumin. Nat Rev Nephrol.

[10] Woolf AS, Gnudi L, Long DA (2009). Roles of angiopoietins in kidney development and disease. J Am Soc Nephrol.

[11] Kim KT, Choi HH, Steinmetz MO (2005). Oligomerization and multimerization are critical for angiopoietin-1 to bind and phosphorylate Tie2. J Biol Chem.

[12] Brindle NP, Saharinen P, Alitalo K (2006). Signaling and functions of angiopoietin-1 in vascular protection. Circ Res.

[13] Suri C, Jones PF, Patan S (1996). Requisite role of angiopoietin-1, a ligand for the TIE2 receptor, during embryonic angiogenesis. Cell.

[14] Jeansson M, Gawlik A, Anderson G (2011). Angiopoietin-1 is essential in mouse vasculature during development and in response to injury. J Clin Invest.

[15] Gavard J, Patel V, Gutkind JS (2008). Angiopoietin-1 prevents VEGF-induced endothelial permeability by sequestering Src through mDia. Dev Cell.

[16] Thurston G, Suri C, Smith K (1999). Leakage-resistant blood vessels in mice transgenically overexpressing angiopoietin-1. Science.

[17] Maisonpierre PC, Suri C, Jones PF (1997). Angiopoietin-2, a natural antagonist for Tie2 that disrupts in vivo angiogenesis. Science.

[18] Hakanpaa L, Sipila T, Leppanen VM (2015). Endothelial destabilization by angiopoietin-2 via integrin beta1 activation. Nat Commun.

[19] Findley CM, Cudmore MJ, Ahmed A, Kontos CD (2007). VEGF induces Tie2 shedding via a phosphoinositide 3-kinase/Akt dependent pathway to modulate Tie2 signaling. Arterioscler Thromb Vasc Biol.

[20] Yuan HT, Suri C, Yancopoulos GD, Woolf AS (1999). Expression of angiopoietin-1, angiopoietin-2, and the Tie-2 receptor tyrosine kinase during mouse kidney maturation. J Am Soc Nephrol.

[21] Satchell SC, Harper SJ, Tooke JE, Kerjaschki D, Saleem MA, Mathieson PW (2002). Human podocytes express angiopoietin 1, a potential regulator of glomerular vascular endothelial growth factor. J Am Soc Nephrol.

[22] Yuan HT, Suri C, Landon DN, Yancopoulos GD, Woolf AS (2000). Angiopoietin-2 is a site-specific factor in differentiation of mouse renal vasculature. J Am Soc Nephrol.

[23] Long DA, Woolf AS, Suda T, Yuan HT (2001). Increased renal angiopoietin-1 expression in folic acid-induced nephrotoxicity in mice. J Am Soc Nephrol.

[24] Dessapt-Baradez C, Woolf AS, White KE (2014). Targeted glomerular angiopoietin-1 therapy for early diabetic kidney disease. J Am Soc Nephrol.

[25] Rizkalla B, Forbes JM, Cao Z, Boner G, Cooper ME (2005). Temporal renal expression of angiogenic growth factors and their receptors in experimental diabetes: role of the renin-angiotensin system. J Hypertens.

[26] Davis B, Dei CA, Long DA (2007). Podocyte-specific expression of angiopoietin-2 causes proteinuria and apoptosis of glomerular endothelia. J Am Soc Nephrol.

[27] Nakagawa T, Kosugi T, Haneda M, Rivard CJ, Long DA (2009). Abnormal angiogenesis in diabetic nephropathy. Diabetes.

[28] Babaei S, Teichert-Kuliszewska K, Zhang Q, Jones N, Dumont DJ, Stewart DJ (2003). Angiogenic actions of angiopoietin-1 require endothelium-derived nitric oxide. Am J Pathol.

[29] Predescu D, Predescu S, Shimizu J, Miyawaki-Shimizu K, Malik AB (2005). Constitutive eNOS-derived nitric oxide is a determinant of endothelial junctional integrity. Am J Physiol Lung Cell Mol Physiol.

[30] Nakagawa T (2007). Uncoupling of the VEGF-endothelial nitric oxide axis in diabetic nephropathy: an explanation for the paradoxical effects of VEGF in renal disease. Am J Physiol Renal Physiol.

[31] Zhu J, Sun N, Aoudjit L (2008). Nephrin mediates actin reorganization via phosphoinositide 3-kinase in podocytes. Kidney Int.

[32] Lim HS, Blann AD, Chong AY, Freestone B, Lip GY (2004). Plasma vascular endothelial growth factor, angiopoietin-1, and angiopoietin-2 in diabetes: implications for cardiovascular risk and effects of multifactorial intervention. Diabetes Care.

[33] Chen S, Li H, Zhang C (2015). Urinary angiopoietin-2 is associated with albuminuria in patients with type 2 diabetes mellitus. Int J Endocrinol.

[34] Tsai YC, Chiu YW, Tsai JC (2014). Association of angiopoietin-2 with renal outcome in chronic kidney disease. PLoS One.

[35] David S, John SG, Jefferies HJ (2012). Angiopoietin-2 levels predict mortality in CKD patients. Nephrol Dial Transplant.

[36] Cruickshank K, Riste L, Anderson SG, Wright JS, Dunn G, Gosling RG (2002). Aortic pulse-wave velocity and its relationship to mortality in diabetes and glucose intolerance: an integrated index of vascular function?. Circulation.

[37] Chang FC, Chiang WC, Tsai MH (2014). Angiopoietin-2-induced arterial stiffness in CKD. J Am Soc Nephrol.

[38] Saharinen P, Alitalo K (2011). The yin, the yang, and the angiopoietin-1. J Clin Invest.

[39] Dei Cas A, Gnudi L (2012). VEGF and angiopoietins in diabetic glomerulopathy: how far for a new treatment?. Metabolism.

[40] Gnudi L, Benedetti S, Woolf AS, Long DA (2015). Vascular growth factors play critical roles in kidney glomeruli. Clin Sci (Lond).

[41] Campochiaro PA, Sophie R, Tolentino M (2015). Treatment of diabetic macular edema with an inhibitor of vascular endothelial-protein tyrosine phosphatase that activates Tie2. Ophthalmology.

